# Evaluating social network-based weight loss interventions in Chinese population: An agent-based simulation

**DOI:** 10.1371/journal.pone.0236716

**Published:** 2020-08-03

**Authors:** Liuyan Shi, Liang Zhang, Yun Lu

**Affiliations:** School of International Pharmaceutical Business, China Pharmaceutical University, Nanjing, Jiangsu, China; Universidad de Burgos, SPAIN

## Abstract

**Objective:**

The aim of this study is to assess network-based weight loss interventions in the Chinese setting using agent-based simulation.

**Methods:**

An agent-based model incorporating social, environmental and personal influence is developed to simulate the obesity epidemic through an interconnected social network among a population of 2197 individuals from the nationally representative survey. Model parameters are collected from literature and existing database. To ensure the robustness of our findings, the model is validated against empirical observations and sensitivity analyses are performed on calibrated parameters.

**Results:**

When compared with the baseline model, significant weight difference is detected using paired samples t tests for network-based intervention strategies (p<0.05) but no difference is observed for the two conventional intervention strategies including choosing random or high-risk individuals (p>0.05). Targeting the most connected individuals minimizes the average population weight, average BMI, and generates a reduction of 2.70% and 1.38% in overweight and obesity prevalence.

**Conclusions:**

The simulations shows that targeting individuals on the basis of their social network attributes outperforms conventional targeting strategies. Future work needs to focus on how to further leverage social networks to curb obesity prevalence and enhance interventions for other chronic conditions using agent-based simulation.

## Introduction

Worldwide, obesity prevalence has increased dramatically over the past few decades [1]. It was reported that the number of individuals with overweight and obesity increased by 27.5% for adults and 47.1% for children from 1980 to 2013 globally [2]. As an important risk factor for several chronic conditions including cancer development [3], overweight and obesity were estimated to cause 3.4 million deaths, 4% of years of life lost, and 4% of disability-adjusted life-years (DALYs) globally in 2010 [4].

Considering the growing prevalence of obesity and related chronic diseases, governments have launched various anti-obesity programs mainly focusing on the first line strategy, that is, lifestyle changes involving decreased energy intake and increased physical activity [5–7]. With about one fifth of people with overweight or obesity in the world, Chinese government has designed policies and guidelines to curb the rapid growth of overweight and obesity [8]. Notably, different from the criteria for overweight and obesity from World Health Organization (WHO) which defines overweight as a body mass index (BMI) between 25 kg/m^2^ and 30kg/m^2^, obesity a BMI of 30 kg/m^2^ and above, the official Chinese definition stipulates overweight as a BMI between 24 kg/m^2^ and 28 kg/m^2^; obesity a BMI of 28 kg/m^2^ and above due to the elevated risk for Chinese population to develop obesity-related diseases at a lower BMI [9]. Nevertheless, no national success against obesity has been reported yet [10].

Although the benefit of weight loss and potential intervention strategies have been studied extensively, few researches have centered on the optimal target population to maximize the effectiveness and cost-effectiveness of anti-obesity interventions. Conventionally, lifestyle interventions are targeted at patients diagnosed with obesity. Generalized messages about weight loss, such as anti-obesity slogans in the television, tend to choose target population at random. However, recent studies indicates that social networks, the complex webs of social relationships and social interactions that connect individuals, play an important role in the development of obesity through peer influence on people’s energy intake and physical activity [11]. Yet there remains an inferential leap from evidence suggesting the influence of social network on obesity development to confidence that leveraging social network to target anti-obesity resources can increase the effectiveness of anti-obesity interventions.

Increasingly, agent-based models (ABMs), a type of computerized simulations of real world dynamic patterns of adaptive behavior, are employed in population health research [12]. ABMs are particularly effective when studying the dynamic interplay between individual level behaviors and population level outcomes [13]. Several ABMs have been developed to understand the social network effect on the obesity epidemic, with the majority simply focusing on the direct spread of the body weight (BW) [14, 15]. Giabbanelli et al. proposed a model in which individuals are not directly acting on others’ body weights but instead influencing the physical activity and energy intake, the imbalance of which subsequently results in changes in body weight [15].

This paper aims to assess network-based weight management interventions for the first time in the Chinese setting using agent-based simulation. It is predicted that exploiting social network for anti-obesity interventions could improve the overall population effectiveness. We also predict that targeting interventions on the most highly connected individuals could maximize the spillover of weight loss effects over the population.

## Methods

### Model structure

The model of obesity epidemic in our research is built on a previously validated model proposed by Giabbanelli et al. [15], which explains the change in body weight through the imbalance of physical activity and energy intake over time and accounts for the interaction of social networks with environmental factors [16]. Since social communities often form a scale-free network, agents are nested in such a social network which is generated by employing a biased preferential attachment growth model and follows the power law degree distribution and possesses homophily properties. The formation of the network is governed by network density and homophily. In each step, the network density determines whether a link is created between existing nodes or a new node is added to the network. Agents with the same gender, and similar age and BW are preferentially connected according to the homophily principle which structures network ties of all kinds of relationship [17].

Individuals’ physical activity (PA) and energy intake (EI) are affected by their neighbors and the environment. The social network influence is determined by the number of individual’s neighbors and the difference of their PA or EI. Specifically, the influence of social network on the EI and PA of a person *i* at the time step t, represented by $Inf_{EI_{i}(t)}$ and $Inf_{PA_{i}(t)}$ respectively, is quantified by the equations below:

$$Inf_{EI_{i}(t)}=\frac{1}{N_{j}}\sum_{j\in F_{i}}\left(EI_{j}(t-1)-EI_{i}(t-1)\right)$$

$$Inf_{PA_{i}(t)}=\frac{1}{N_{j}}\sum_{j\in F_{i}}\left(PA_{j}(t-1)-PA_{i}(t-1)\right)$$

where *j* are the individuals connected to *i*. *F*_*i*_ and *N*_*j*_ denote the set and the number of those individuals.

The environmental influence (Env) comes from sources such as advertising, education, and the built environment and is set to be a random value between 0 to 2, which represents a harmful environment when 0 < Env < 1 or a beneficial one when 1 < Env < 2. When the social network influence is positive, a beneficial environment will reduce the effect while a harmful one will further increase it. Thus the combined influence of social network and the environment on the EI of a person *i* at the time step t ($Inf_{EI_{i}(t)Env}$) is defined as follows:

$$if\;Inf_{EI_{i}(t)}\geq 0,\;\mathrm{then}\;Inf_{EI_{i}(t),Env}=\frac{1}{\mathrm{Env}}Inf_{EI_{i}(t)}$$

$$if\;Inf_{EI_{i}(t)}<0,\;\mathrm{then}\;Inf_{EI_{i}(t),Env}=\mathrm{Env}Inf_{EI_{i}(t)}$$

The effect on the PA is the opposite and thus the combined influence of social network and the environment on the PA of a person *i* at the time step t ($Inf_{PA_{i}(t)Env}$) is estimated as follows:

$$if\;Inf_{PA_{i}(t)}\geq 0,\;\mathrm{then}\;Inf_{PA_{i}(t),Env}=\mathrm{Env}Inf_{PA_{i}(t)}$$

$$if\;Inf_{PA_{i}(t)}<0,\;\mathrm{then}\;Inf_{PA_{i}(t),Env}=\frac{1}{\mathrm{Env}}Inf_{PA_{i}(t)}$$

If the combined influence of social network and the environment exceeds the threshold fixed for all agents, or is lower than the minus of the threshold, the agent would change its PA or EI accordingly.

$$if\;{Inf}_{EI_{i}(t),Env}>T_{EI}EI_{i}(t-1),\;\mathrm{then}\;EI_{i}(t)=EI_{i}(t-1)+I_{EI}EI_{i}(t-1)$$

$$if\;Inf_{EI_{i}(t),Env}<‐T_{EI}EI_{i}(t-1),\;\mathrm{then}\;EI_{i}(t)=EI_{i}(t-1)‐I_{EI}EI_{i}(t-1)$$

where T_EI_ represents the threshold for EI, I_EI_ the impact on EI.

$$if\;{Inf}_{PA_{i}(t),Env}>T_{PA}PA_{i}(t-1),\;\mathrm{then}\;PA_{i}(t)=PA_{i}(t-1)+I_{PA}PA_{i}(t-1)$$

$$if\;{Inf}_{PA_{i}(t),Env}<‐T_{PA}PA_{i}(t-1),\;\mathrm{then}\;PA_{i}(t)=PA_{i}(t-1)‐I_{PA}PA_{i}(t-1)$$

where T_PA_ represents the threshold for PA, I_PA_ the impact on PA.

The change in PA and EI results in the imbalance of energy expenditure (EE) and EI, which is transformed to body weight (BW) change through energy density that is determined by a formula with regard to the initial BW. We consider that individuals adjust their PA an EI every seven days according to the influence they subjected to. In view of the fact that daily routine is difficult to break, we assume that individuals change their PA and EI gradually instead of suddenly, thus causing a discount (λ) on the energy imbalance within each cycle.

### Model parameters

Information on age, gender, height, BW and EI from the database and network density from the literature are used as personal attributes and attribute of the social network respectively to design an agent-based social network model of people in China in 2009. We borrow information in the context of Chinese community from the literature to set the network density to 0.267 [18]. The individual threshold and environmental influence are obtained by fitting the model to the China Health and Nutrition Survey (CHNS) database and calibrated by sensitivity analysis. The T_EI_ is 0.07, T_PA_ is 0.12, and Env ranges from 0.82 to 1.08.

As is mentioned above, each agent is assigned the personal attributes including age, gender, height, BW and EI from the CHNS database. To determine the total energy expenditure (TEE), we assume that all individuals are in an equilibrium state, that is, they are not gaining or losing weight during the 2009 CHNS survey which investigated the 3-day EI of respondents. The TEE in humans is composed of three components [19]. The resting energy expenditure (REE), which sustains the basic functions of the body such as resting cardiopulmonary activity, accounts for approximately 60 to 75 percent of TEE [20]. The thermic effect of food (TEF) is the energy expended in processing food intake and accounts for approximately 10 percent. The effect of PA is the energy used to support muscular work. To estimate the PA, we first calculate the TEF through its percentage of the TEE and then the REE based on the equations below [21]:

Formales:REE=66.5+13.6*BW+5.0*Height−6.8*Age

Forfemales:REE=655.1+9.5*BW+1.8*Height−4.1*Age

### Data sources

The sample is extracted from the CHNS database, which is an ongoing open cohort, international collaborative project between the Carolina Population Center at the University of North Carolina and the National Institute for Nutrition and Health at the Chinese Center for Disease Control and Prevention. The database contains detailed demographic characteristics and health indicators of over 30,000 individuals in 15 provinces and municipal cities.

**Fig 1:**
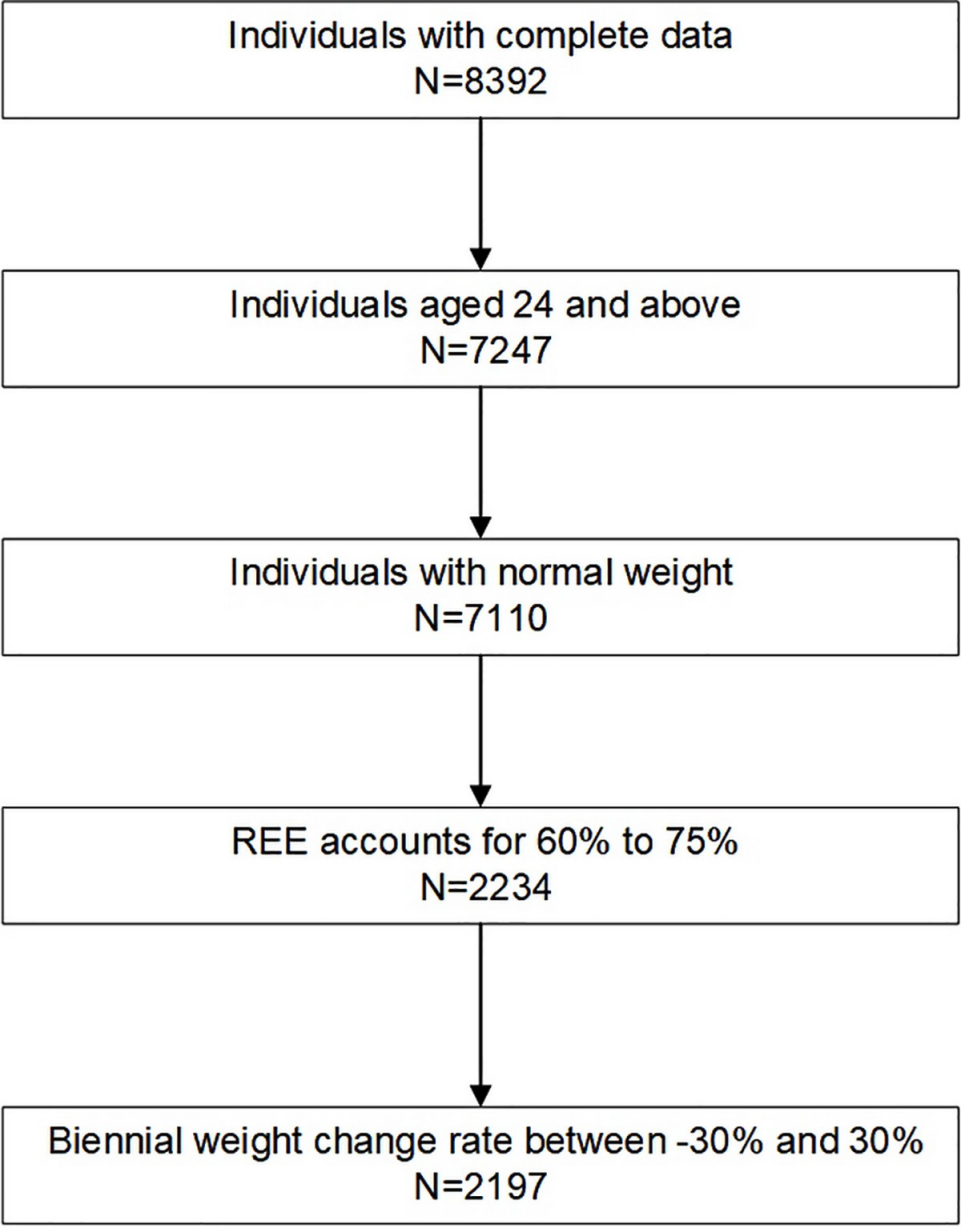
The selection process of simulated agents. Individuals with complete and reasonable data are included. REE, resting energy expenditure.

As discussed above, information on the personal attributes of individuals including age, gender, height, body weight and energy intake at Wave 8 and body weight at Wave 9 is necessary so that only a total of 8,392 individuals with all those data are included in the study by far. Since the heights of individuals were assumed to be constant, respondents aged under 24 (1145) who were with unclosed epiphyseal plate and likely to grow are excluded [22]. In addition, we exclude respondents with unusual BW, EE, and biennial BW change to ensure the authenticity of the sample. In the remaining respondents, individuals whose body weight was abnormal (137), that is, being 0 or 5.8 kg, are also excluded from the sample. Under normal circumstances, the REE accounts for approximately 60 to 75 percent of the TEE. Therefore, individuals whose REE is under 60 percent or above 75 percent of the TEE (4876) because of mistakes in recalling or recording are excluded. Furthermore, statistical analysis of the remaining 2,234 individuals showed that 98.3% of them had a two-year weight change rate of -30% to 30%. Therefore, individuals with a weight change rate outside the range are considered to have extreme body weight change due to diseases or mistakes in recording and are also not included in the study. The inclusion criteria select 2,197 people with complete and reasonable data to be included in the study. Fig 1 shows the selection process of the sample.

### Simulations and validation

The obesity epidemic model is initiated by the data from Wave 8 (2009) of the CHNS database and is validated against the weight of participants at Wave 9 (2011). We compare the effect of obesity interventions targeting five different populations selected by two conventional methods and three network-based methods. Conventional methods target interventions randomly (hereby referred to as Random) or at population with overweight or obesity (hereby referred to as High-risk). Network-based methods choose targeted individuals with highest degree, betweenness, or closeness centrality (hereby referred to as Degree, Betweenness, Closeness respectively) [23, 24]. Agents with highest degree centrality are individuals with the largest number of connections and therefore interact most frequently with their neighbors and occupy the central position of the network. The betweenness centrality measures the level that the agent controls the resources. Agents with high betweenness centrality affect the interaction between other pairs of agents to some extent, and possess more interpersonal influences. If the shortest distance between an agent and all the other agents is the smallest, the agent is with highest closeness centrality. Such agents can quickly connect with other nodes so they are able to efficiently exchange information with other agents. The agents with high degree, betweenness, and closeness centrality are selected using functions of their number of connections or position in the network, which are calculated automatically using Netlogo as attributes of agents nested in a network.

The conventional and network-based interventions are implemented among 5% of the population each week, causing a decrease of 5% in EI or an increase of 5% in PA. To ensure the robustness of our findings, we run further simulations without intervention (the control simulation) for two years to compare the results against the empirical observations at Wave 9. Simulations with those five different weight loss interventions are also run for 2 years and the results are compared with those of the baseline model and the control simulation to evaluate the intervention effectiveness.

The simulation platform used in the study is Netlogo 6.0.4 (Wilensky, 1999), and the data analysis software is Microsoft Office Professional Plus 2013 for Windows and Stata softwareV.14.0 for Windows (Stata Corp). All results are the average of 100 independent runs of the model and are compared with those of the control simulation.

## Results

The sample includes 2197 respondents aged 24 to 88, 44.9% of whom is male. The height and BW of individuals from the sample ranges from 1.30 to 1.88 m, 29.5 to 109.2 kg respectively. The average BMI is 24 kg/m^2^, with 805 (36.6%) respondents being overweight and 285 (13.0%) being obese.

The effectiveness of five intervention strategies is evaluated by comparison with the baseline model and among different strategies using outcome measures including BWs of individuals, the average BW, average BMI, overweight prevalence, and obesity prevalence of the population. To ensure the robustness of our results, the model is validated against empirical observations and a set of sensitivity analysis is conducted on the four calibrated parameters.

### Model validation results

**Fig 2:**
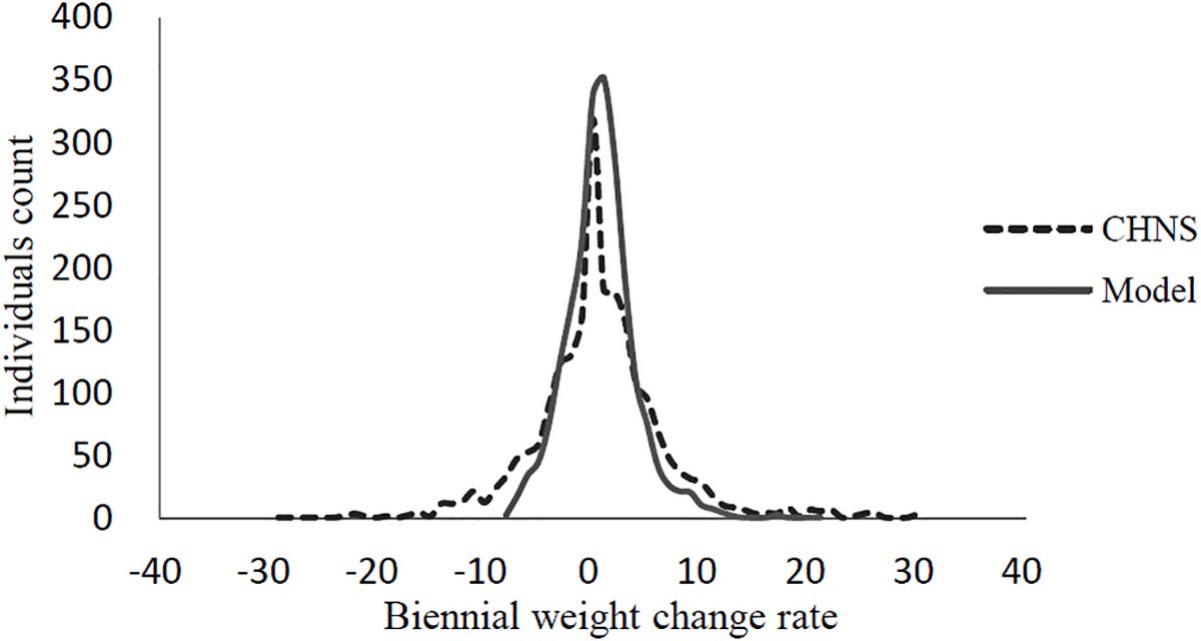
Comparison of the biennial weight change distribution between our simulations and the CHNS database.

The model is first run for two years without any intervention. The average weight change of the population over the two years was 0.2 kg with a standard deviation of 1.9 kg, comparable with 0.3 kg and 3.9 kg in the CHNS database. At the individual level, a paired samples t test is performed to test for the weight differences between participants in the model and the CHNS database and the results shows no significant difference between the two groups (p>0.05). Fig 2 presents comparison of the biennial weight change distribution between our simulations and the CHNS database.

### Effect of obesity intervention strategies on the individual weight

When compared with the baseline model, significant weight difference is detected using paired samples t tests for the three network-based intervention strategies (p<0.05) but no difference is observed for the two conventional intervention strategies (p>0.05). As for the comparison with the control simulation, paired samples t tests demonstrates that there is significant difference in weight of individuals for all five intervention strategies (p<0.05).

### Effect of obesity intervention strategies on the average population weight

**Fig 3:**
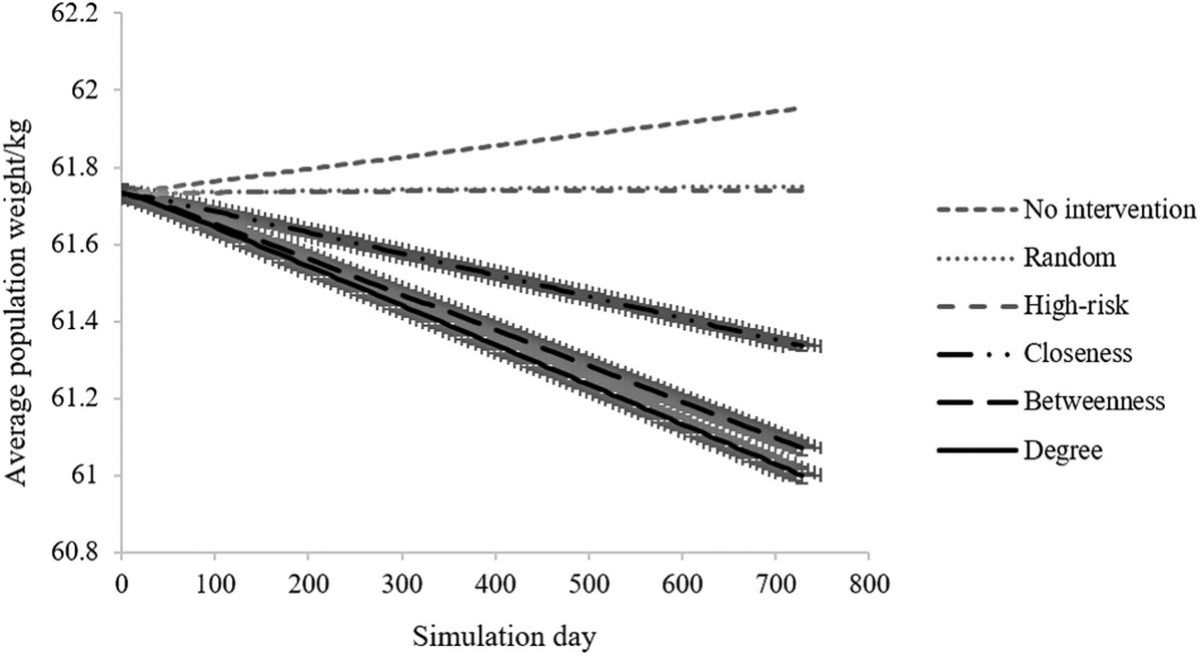
The average population weight over the two years in different intervention scenarios. The control simulation without any intervention is also shown to compare with the other intervention strategies. No-intervention, the control simulation without any intervention; Random, intervention strategy targeting individuals at random; High-risk, intervention strategy targeting individuals with overweight or obesity; Closeness, intervention strategy targeting individuals with highest closeness centrality; Betweenness, intervention strategy targeting individuals with highest betweenness centrality; Degree, intervention strategy targeting individuals with highest degree centrality. The standard error of the Degree method is shown in the figure.

The average population weight over the two years in different intervention scenarios is plotted in Fig 3. The dashed line represents the average network weight change of the control simulation. Consistent with the empirical observations, the average weight of the population increases slightly (0.22 kg; 95%CI, 0.21–0.24 kg) without any intervention. Compared with the control simulation, the five intervention approaches induce decreases in the average weight within the range between 0.21 kg (95%CI, 0.19–0.22 kg) and 0.96 kg (95%CI, 0.95–0.98 kg). Generally speaking, targeting individuals based on their network position generates larger decline in average population weight. Targeting individuals with the most connections yields the best effect on the average population weight, with a decrease of 0.96 kg (95%CI, 0.95–0.98 kg) in average weight, followed by 0.89 kg (95%CI, 0.87–0.90 kg) and 0.62 kg (95%CI, 0.61–0.64 kg) after interventions targeting individuals with the highest betweenness and closeness centrality. The two conventional interventions perform similarly, with a total network weight loss of 0.21 kg (95%CI, 0.19–0.22 kg) and 0.22 kg (95%CI, 0.20–0.23 kg), respectively.

### Effect of obesity intervention strategies on the average population BMI

The five intervention strategies exerts similar effects on the average BMI of the population and the average network weight. Compared with the control simulation, the average BMI of the population decreases after all the five interventions. The two conventional interventions reduces the average BMI less than the three network-based interventions, among which targeting individuals with the highest degree centrality has the strongest influence, causing the average population BMI decreased by 0.37 kg/m^2^. The intervention strategy of targeting individuals with the highest degree centrality is followed by targeting individuals with the highest betweenness and closeness centrality, which lead to a reduction of 0.34 kg/m^2^ and 0.24 kg/m^2^ in the average BMI respectively. Both conventional interventions shows the least overall impact and reduces the average BMI by 0.08 kg/m^2^.

### Effect of obesity intervention strategies on overweight and obesity prevalence

Overweight and obesity rates are also computed to reflect the effectiveness of obesity interventions. Table 1 shows the prevalence of overweight and obesity after different intervention strategies. All of the five weight management interventions have a positive effect in reducing overweight and obesity prevalence of the simulated population. The percentage of obesity individuals drops to less than 10% after network-based interventions over two years while conventional interventions yields a percentage higher than 10%. Again the best performing strategy is targeting the best connected individuals, which generates an average reduction of 2.70% and 1.38% in overweight and obesity prevalence.

**Table 1:** The prevalence of overweight and obesity after different intervention strategies.

Intervention strategy	Overweight rate/mean ± SD	Obesity rate/mean±SD
**No-intervention**	34.07±0.51	10.64±0.31
**Random**	33.55±0.57	10.31±0.33
**High-risk**	33.31±0.55	10.02±0.29
**Closeness**	32.22±0.58	9.88±0.32
**Betweenness**	31.65±0.54	9.32±0.29
**Degree**	31.37±0.58	9.26±0.29

SD, Standard Deviation; the other abbreviations have been explained in Fig 3.

### Sensitivity analyses on the calibrated parameters

**Fig 4:**
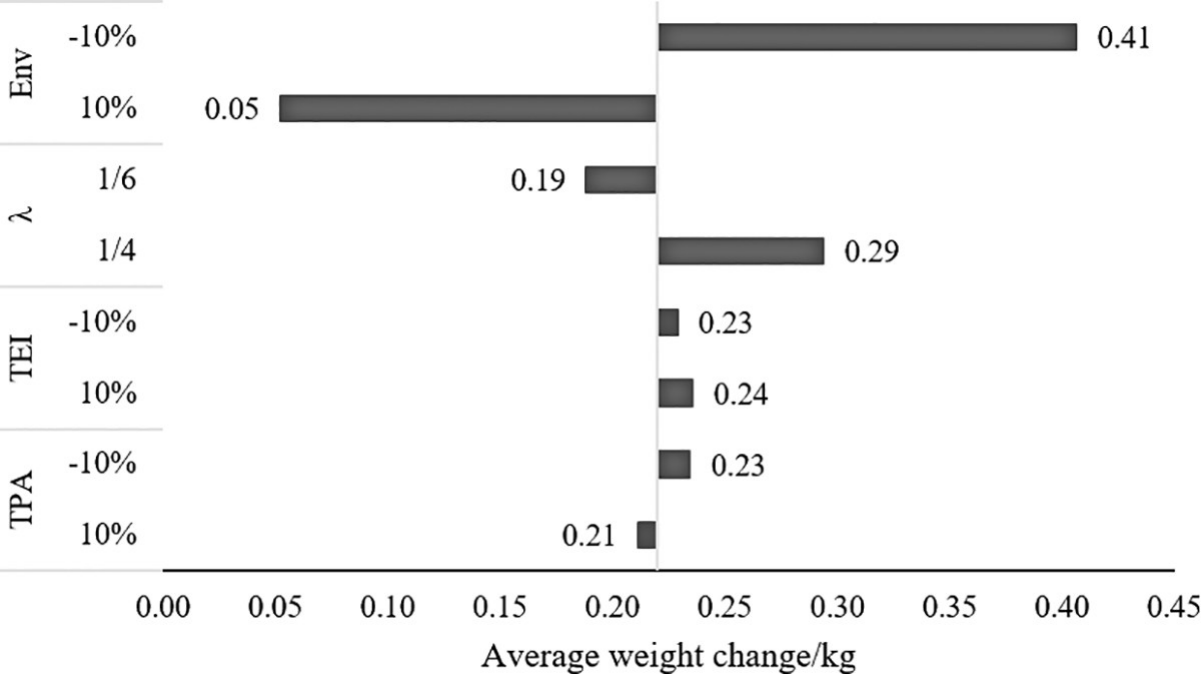
Sensitivity analyses results. The vertical axis represents the adjusted parameter and the horizontal axis is the corresponding average weight change of the population. The intersection point is the baseline without any intervention. T_EI,_ threshold for energy intake; T_PA,_ threshold for physical activity; Env, environmental influence.

To test the robustness of the simulation results, sensitivity analyses are performed on the four calibrated parameters including T_EI_, T_PA_, λ, and Env. In the baseline model, the four parameters are configured as λ = 1/4, T_EI_ = 0.07, T_PA_ = 0.12, Env ranging from 0.82 to 1.08, resulting in an increase of 0.22 kg in the average weight of the entire population. At each time, one of the four parameters is adjusted by 10% or -10% of its initial value. The average network weight changes ranges from 0.05kg to 0.41kg and are presented in Fig 4.

## Discussion

The present study is the first to evaluate network-based obesity interventions in China. The spread of obesity is simulated through an interconnected social network among a population of 2197 individuals from the nationally representative survey. As we predict, the results indicates that network-based targeting methods lead to greater population effectiveness in anti-obesity interventions. To be specific, targeting individuals with the most connections results in the largest reduction in the average BW and BMI of the population, obesity rate and overweight rate.

This study adds to a little but growing literature that attempts to determine the most effective weight loss strategies in a networked population using agent-based simulation. Bahr et al. used the 2003–2004 distribution of BMIs in the United States to initiate their social network [14]. Afterwards, the BMIs were updated at each time step following the rules that an individual’s weight gain was positively correlated with that of their contacts. Regardless of the topology of networks, the simulations demonstrated that individuals with similar BMIs would cluster together and the most highly connected individuals had a disproportionate effect on the population due to its tremendous number of neighbors. The authors thus concluded that one of the most effective weight management approach would be targeting interventions in the best-connected individuals on the edge of a cluster.

The simulations show that targeting individuals on the basis of their social network attributes outperforms conventional targeting strategies including choosing random or high-risk individuals. This is in agreement with some of the previous reports, Hammond et al. modelled social influence on body weight based on evidence from physiology, social psychology, and behavioral science [25]. Their simulations suggested that multiple distinct pathways of social influence contributed to changes in obesity and the dynamic interplay of individual behavior and social norms could potentially be leveraged to yield downward pressure on BMI. However, this result also contrasts with other researches. Zhang et al. incorporated real-world network structure into the simulation of body weight change caused by social network dynamics and peer influence [26]. Their conclusions included that the effect of peer influence depended on the distribution of BMI and in high-obesity populations, enhanced peer influence might increase the prevalence of overweight and obesity.

The best performing intervention strategy in our study is targeting the most connected individuals, which minimizes the average population weight, average BMI, overweight prevalence and obesity prevalence. This is consistent with some of the prior researches. Trogdon et al. simulated the peer selection effect on social multiplier for anti-obesity interventions and identified that targeting interventions on the most popular individuals with obesity maximized the spillover of body weight loss [27]. Therefore, they concluded that considering network-based attributes could improve the effect of anti-obesity interventions. However, other studies drew the opposite conclusion. El-Sayed et al. simulated the obesity epidemic in a densely connected social network among 10,000 individuals representing an English cohort [28]. After comparing the effectiveness of targeting obesity preventive and treatment interventions on the best connected agents with those targeting agents randomly, the authors found no difference in the progression of obesity between targeting the most connected individuals and targeting individuals at random.

There are several limitations to this study that the readers should be aware. One limitation of the research is the failure to build a network with full fidelity to reality due to lack of data on social networks including the number and strength of connections among individuals within the same community. Consequently, a mixture of scale-free mechanisms and homophily is used to generate the network in our study, which is considered to be close to real-world networks. This research is also limited by the restricted number of network-based intervention strategies. It is plausible that there are other interventions left untested which could produce different results. A third limitation is that our agent-based model is parameterized using data from Chinese context. Therefore, our findings may not generalize to other populations.

In spite of these limitations, our findings have important implications for public policy and future research. From the perspective of policymakers, our research provides evidence that targeting the most highly connected individuals could improve the effectiveness of weight loss interventions compared with targeting individuals at random or at risk. From the perspective of researchers, our study employs agent-based simulation to analyze the underlying mechanism for the obesity epidemic and social dynamics, both of which are underexplored fields of research in China partly due to their uncertainty and complexity. Future work may consider using this technology to leverage social networks to curb obesity prevalence and enhance interventions for other chronic conditions.

## Supporting information

S1 Data The information on the personal attributes of individuals from the China Health and Nutrition Survey (CHNS) database. (XLSX)

S1 Model The obesity epidemic and intervention model. (ZIP)
